# Exploring Human Misuse and Abuse of Veterinary Drugs: A Descriptive Pharmacovigilance Analysis Utilising the Food and Drug Administration’s Adverse Events Reporting System (FAERS)

**DOI:** 10.3390/toxics12110777

**Published:** 2024-10-25

**Authors:** Josie Dunn, Fabrizio Schifano, Ed Dudley, Amira Guirguis

**Affiliations:** 1 Medical School, The Grove, Swansea University, Singleton Campus, Wales SA2 8PP, UK; 2009986@swansea.ac.uk (J.D.); e.dudley@swansea.ac.uk (E.D.); 2 Psychopharmacology, Drug Misuse and Novel Psychoactive Substances Research Unit, School of Life and Medical Sciences, University of Hertfordshire, Hatfield AL10 9AB, UK; f.schifano@herts.ac.uk

**Keywords:** veterinary medicines, animal medicines, substance use, pharmacovigilance, diversion of medicines, drug misuse

## Abstract

Introduction: Evidence suggests an increasing misuse of veterinary medicines by humans. This study aims to analyse Adverse Events (AEs) associated with selected veterinary products using the Food and Drug Administration Adverse Events Reporting System (FAERS). Methods: A descriptive pharmacovigilance analysis was conducted on AEs related to 21 drugs approved for human and/or animal use. Results: A total of 38,756 AEs, including 9566 fatalities, were identified. The United States reported the highest number of cases (13,532), followed by Canada (2869) and the United Kingdom (1400). Among the eight drugs licenced exclusively for animals, levamisole, pentobarbital, and xylazine were most frequently reported. Reports predominantly involved males (57%) from the 18–64 age group, with incidents related mainly to overdose, dependence, and multi-agent toxicities. Unmasking techniques revealed ‘intentional overdose’ as the primary reaction. Polysubstance use was evident in 90% of the drugs, with benzodiazepines/Z-drugs and opioids as common co-used classes. Conclusions: Veterinary medications are increasingly infiltrating the illicit drug market due to their pharmacological properties. This trend highlights the need for heightened vigilance and awareness to prevent further public health risks associated with the adulteration of illicit substances with veterinary products like xylazine and pentobarbital.

## 1. Introduction

The problem of drug misuse and its contribution to the rising number of drug-related deaths has been a recognised issue for the last few decades. Official statistics show that drug-related deaths in England and Wales have risen for the 11th year in a row, reaching their highest since records began in 1993. However, it has been noted that prescription and over-the-counter medication misuse is a significantly under-recognised problem, affecting a range of vulnerable individuals [1,2]. However, it has been noted that prescription and over-the-counter medication misuse is a significantly under-recognised problem affecting a range of vulnerable individuals [3]. Dependence on prescription drugs, particularly opioids and other controlled substances, represents an increasing public health and clinical challenge in the United Kingdom (UK) and internationally [3]. This growing dependency drives individuals to explore innovative and often risky methods to satisfy their need for these drugs. With more drugs of misuse being controlled and recognised, veterinary medication misuse has become an emerging issue. It is unclear why people who use drugs (PWUD) have increasingly turned to veterinary medications, but it has been observed that most prescription drug misuse is facilitated through healthcare providers [4]. Given that veterinary clinics can serve as alternative sources of medications, they may become targets for those seeking to misuse prescription drugs outside traditional healthcare channels.

Data on veterinary prescription drug misuse is sparse in the UK; however, in the United States (US), these medications have increasingly been recognised as sources of misuse, primarily due to easy access and availability [5]. Veterinarians are frequently overlooked as potential contributors to prescription drug misuse [6]. However, there is a growing interest in obtaining medications through veterinarians, due to the perception of them being safer and cheaper than street drugs [7]. Between 2014 and 2019, it was documented that the number of patients, presumed to be animals based on age data, with prescriptions for any class of controlled substances in the US from four or more veterinarians, increased 3-fold [8]. Additionally, reports indicate that individuals harmed their pets to obtain veterinary analgesics [9]. As veterinarians do not fall under the same monitoring and control constraints as general practitioners, there is potential for increased opportunities for drug diversion and misuse. A study documented that 75% of a sample of veterinarians were aware of working with someone with a substance abuse problem [10].

In addition to users obtaining veterinary drugs directly through clinics, there has also been a rise in adulteration of common drugs of misuse with veterinary products, mainly heroin and fentanyl. Veterinary medicines, including xylazine, carfentanil, dexmedetomidine, pentobarbital, and levamisole, have all been identified in seized fentanyl tablets in the US [11]. This poses a significant concern as users may unknowingly ingest these veterinary substances, leading to potentially serious harm. In 2023, it was reported by the Centres for Disease Control and Prevention (CDC) that the monthly percentage of deaths involving xylazine, a veterinary tranquiliser, in the context of illicitly manufactured fentanyl (IMF), increased by 276%, rising from 2.9% in January 2019 to 10.9% in June 2022 [12]. The escalating fatalities associated with xylazine misuse in the US raised concerns that a similar trend may emerge in the UK, akin to the opioid crisis. As polydrug consumption increases, the challenges of developing effective responses to reduce drug overdose deaths and drug-related poisonings rise [13], where mixtures containing novel opioids and benzodiazepines (BZDs) have been found to contain xylazine. This increase in diversity in drug supply was described by the European Monitoring Centre for Drugs and Drug Addiction (EMCDDA) to pose new challenges for drug policy and healthcare in Europe, with these mixtures having the potential to impact European health [14]. Xylazine use has been increasing rapidly over the last few years, causing the US to announce a public safety alert in 2022 and an announcement in 2023 describing it as an “emerging threat”—with this type of report being a first in US history [15,16]. Although xylazine deaths remain significantly lower in the UK, there is evidence that xylazine has been detected in the UK illicit drug supply, with eleven fatalities documented between May 2022 and August 2023 [17]. Ketamine and carfentanil are two other popular veterinary medications to be misused by humans, with both these drugs being listed as veterinary products with significant health hazards to human health [18]. The latest Focal Report on the UK drug situation in 2019 reported that ketamine usage has reached its peak, at 0.8% [19], with a simultaneous rise in deaths attributed to recreational ketamine misuse [20]. Carfentanil’s misuse is also believed to be under-reported, primarily because it is excluded from most routine drug screenings. Additionally, its dosage regimens and abuse liability remain unclear as it is not licenced for humans [21]. Despite the lack of data regarding carfentanil, 92% of syringes collected in Lithuania contained carfentanil [22].

To the best of our knowledge, there is no current study that investigated veterinary misuse using pharmacovigilance approaches informed by a systematic literature review. Therefore, this study aims to analyse the Food and Drug Association Adverse Events Reporting System (FAERS) for Adverse Events (AEs) associated with selected veterinary products that have been identified from a systematic literature review.

## 2. Materials and Methods

### 2.1. Prior Research

A systematic literature review (SLR) exploring the confluence of animal medicine and its implications for human health was conducted by the research group to investigate which specific veterinary medications are currently being misused by humans [23]. The review searched academic databases such as PubMed, Scopus, and Web of Science, using keywords such as “veterinary drug” OR “veterinary medication” OR “veterinary prescription drug” AND “misuse” OR “abuse”. Inclusion criteria focused on studies that provided evidence of veterinary drug misuse or diversion to human use. Results from this study found 28 distinct veterinary products, 15 that are exclusively for animal use and the remaining 13 approved for use in both humans and animals. Drugs licenced and approved for animal use only that were found to be misused by humans included xylazine, medetomidine, Telazol, carfentanil, pentobarbital, acepromazine, levamisole, tilmicosin, embutramide/mebezonium, dinoprost, cloprostenol, phenylbutazone, flunixin, carprofen, and the vitamin ADE compound. Drugs approved for use in animals and humans included dexmedetomidine, clenbuterol, ketamine, tramadol, butorphanol, diazepam, clorazepate, phenobarbital, pheniramine, stanozolol, levothyroxine, furosemide, and amitriptyline. These drugs, while intended for legitimate medical purposes, can be diverted for human misuse or utilised in veterinary-grade doses. Commonly misused drugs like diazepam were identified through the SLR but were excluded from the study due to a lack of evidence for veterinary-grade diazepam misuse or diversion through veterinary clinics for human misuse.

### 2.2. Search Strategy

The FAERS Public Dashboard [24] is an online database, free to use by consumers, healthcare professionals, and manufacturers to report and view adverse events associated with drug products. The database currently has 28,655,483 total reports, dating back to 1968. The year 2022 had the highest number of reports, with a total of 2,338,998. It is important to note that the FAERS is based on the reporting parties’ observations and assessments, which may not include laboratory confirmation or toxicological analysis. However, the reports in the FAERS are evaluated by clinical reviewers to monitor the safety of products after they are approved by the Food and Drug Administration (FDA).

In line with previous studies that have conducted similar analyses of AEs associated with drugs of misuse [25], we employed a pharmacovigilance approach to systematically retrieve and analyse data on AEs associated with these veterinary products. The database encompasses valuable data on AEs, medication errors, and patient demographics, making it a crucial asset for regulatory science [26]. Trends and patterns can be identified as the FDA database contains a vast amount of data that reflects real-world outcomes related to drug use. By combining both the systematic literature review with the FAERS data analysis, novel data can be retrieved regarding the emerging problem of veterinary medicine misuse in humans.

### 2.3. Selected Preferred Terms

In pharmacovigilance, ‘misuse’ denotes the intentional and improper utilisation of a product, diverging from prescribed guidelines, while ‘abuse’ involves the deliberate non-therapeutic use of a product, motivated by presumed rewards [25]. Preferred Terms (PTs) were designated from the Medical Dictionary for Regulatory Activities (MeDRA), a recognised set of terms relating to medical conditions and medicines [27]. Terms selected and deemed relevant for this study were: overdose, intentional overdose, accidental overdose, drug abuse, substance abuse, off-label use, intentional product misuse, product use in an approved indication, prescription drug used without prescription, toxicity to various agents, poisoning, dependence, substance dependence, drug withdrawal syndrome, withdrawal syndrome, drug diversion, completed suicide, suspected suicide, suicide attempt, and suspected suicide attempt. No date limits or geographical limitations were imposed, encompassing AE reports up to Q4 of 2023, with no instances of duplicate cases identified.

### 2.4. Analysis

The drug names used in the FDA database corresponded to those listed in the British National Formulary (BNF) [28]. For the drugs that were not included on the BNF, such as clenbuterol, the selection was based on FDA database entry with the highest number of reports, ensuring the most relevant drug profile was chosen. The parameters analysed include sex, age, indications of use, country of origin, year of report, reporter type, and the outcomes (e.g., hospitalisation, death, life-threatening outcome). To investigate polydrug use, a separate list of commonly misused drugs was created. This list was separated into drug classes (see Supplementary Information (SI) Table S1), including opioids (e.g., hydrocodone, tramadol, and oxycodone), BZDs (e.g., alprazolam, bromazolam, and flunitrazepam), stimulants (e.g., cocaine, amphetamine, and methylphenidate), and central nervous system (CNS) depressants (e.g., alcohol, gamma-hydroxybutyrate (GHB), and medetomidine). Common drugs of misuse that did not align with the drug groups were listed under ‘other drugs of misuse’ (e.g., cannabis, ayahuasca, gabapentin, and promethazine). Common brand names (e.g., Xanax, Adderall, and Ambien) of these drugs were also cross-referenced to ensure comprehensive data collection. Subsequently, these drugs were extracted from the dataset if they were identified as being concurrently used with the drugs of interest in this current study. A descriptive analysis was then conducted to analyse the AEs associated with the veterinary products extracted from the literature. The reporting odds ratio (ROR) for the drugs analysed in this study was not calculated due to their diverse classification across different drug classes. Since the drugs under investigation belonged to varying groups, comparing their adverse event reporting frequencies using ROR may not yield meaningful results. Of all the drugs included in this study, eight drugs (xylazine, pentobarbital, carfentanil, levamisole, acepromazine, tilmicosin, carprofen, and phenylbutazone) are exclusively approved for animals only. Unmasking techniques were employed to analyse the specific effects of the drug of interest in isolation. This involved examining adverse events where no other drugs were identified, thereby isolating the adverse effects attributed solely to the drug in question. By filtering out cases involving polydrug use, this method allowed for a clearer understanding of the drug’s independent impact. Data analyses were performed using Microsoft Excel (Version 16.83 (24031120)).

## 3. Results

### 3.1. Querying the FAERS Database for AEs

The FAERS was queried in January 2024 for AEs related to the veterinary drugs that were retrieved from the systematic literature review [23]. Due to the human-centric nature of the FAERS, data for specific veterinary products were unavailable for analysis as it was not included in the FAERS database. Consequently, some drugs were excluded from this study as no associated AE reports were found. These included the veterinary compound telazol, embutramide/mebezonium, cloprostenol, medetomidine, and the veterinary ADE vitamin compound. Flunixin data were available on the FAERS; however, it did not include data relevant to this study. Diazepam was also not included in this study due to the absence of reports on human misuse of veterinary-grade diazepam diverted from clinics.

### 3.2. Overview of AEs and Mortality

**Figure 1:**
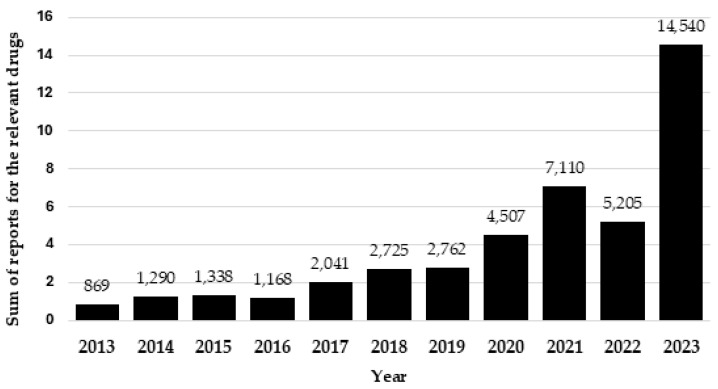
The total number of selected adverse reactions using ‘Preferred Terms (PTs)’ being reported for the drugs included in the study, except carprofen and tilmicosin, per year, over 10 years from 2013 to 2023.

From the 21 drugs that were included in this study, there were a total of 198,640 adverse events reported to the FDA up until 31 December 2023. Among these, 38,756 (20%) adverse events related to the selected PTs were reported for the same 21 drugs. There was a total of 9566 (25%) deaths associated with the PTs for all drugs included in this study. Figure 1 demonstrates the general increase in the number of reports received by the FDA for the last 10 years for the associated drugs. Carprofen and tilmicosin were excluded from this total as they have received no relevant reports in the last decade.

### 3.3. Reporting Trends and Demographics

**Figure 2:**
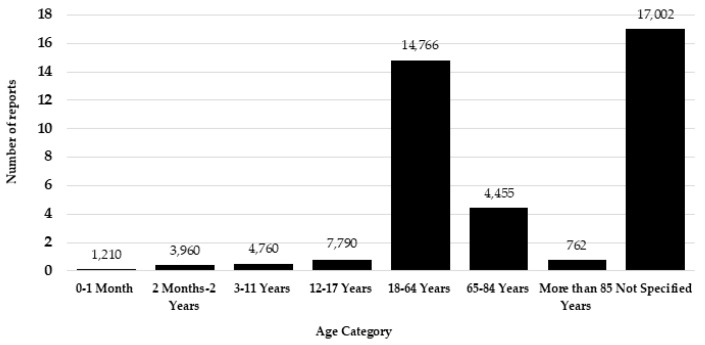
The distribution of reports across different age categories.

Examining the eight drugs exclusively approved for animal use, there is a noticeable overall increase in the number of reports for levamisole, pentobarbital, and xylazine. Carfentanil’s reports to the FAERS peaked in 2021 (41 reports) and slightly decreased in 2022 (19), with only 3 reports in 2023. Acepromazine had the highest number of reports in 2017 (33), yet reports have remained low since then. Phenylbutazone has received just seven reports since 2017, with only one report in 2023. Of the 21 drugs analysed, 12/21 (57%) demonstrated a higher number of reports from males, whereas 9/21 (43%) exhibited a greater number of reports from females. Although more drugs had males as the more common reporter, the total number of reports for all 21 drugs was higher for females. Females contributed to a total of 16,076 (50%) reports, whereas males reported 15,927 (50%) altogether. From the total number of reports, the ‘not specified’ age group accounted for 17,002 reports. Excluding the cases where the age was not classified, there were a total of 21,755 reports that were separated by age group. Of these, the age group 18–64 years accounted for 14,766 (68%) reports. Figure 2 illustrates the distribution of reports across specified age groups. The 18–64 age group had the highest number of reports for 20 out of 21 drugs, with furosemide having the highest number of reports in the 65–84 age category. Healthcare professionals submitted 20,431 (53%) total reports, while consumer reports accounted for 16,804 reports (44%). The reporter type for the remaining 1512 (4%) reports was unspecified. After excluding reports categorised as an ‘unknown outcome’, hospitalisation emerged as the most common outcome with 12,447 reports (44%), followed by death as the secondary outcome with 9566 reports (34%). Non-serious outcomes represented only 5% (1553) of reports.

### 3.4. Adverse Reactions

**Figure 3:**
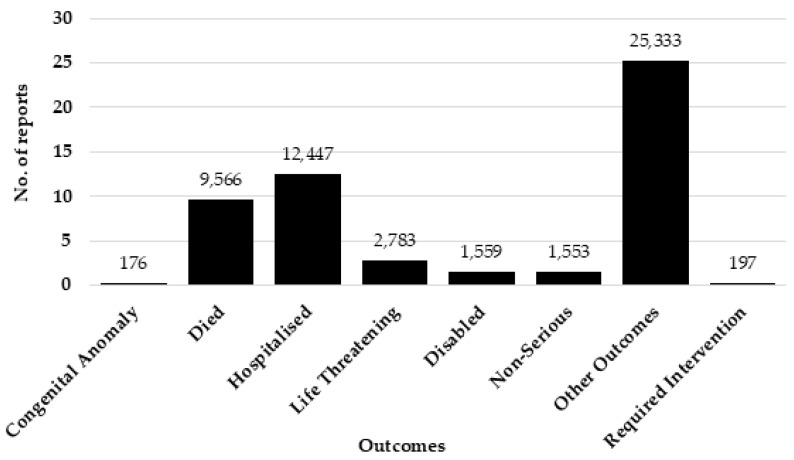
The various outcomes related with the reports.

Of the 21 selected PTs, ‘overdose’ was the most reported reaction, followed by ‘dependence’ and ‘toxicity to various agents’, with reports of 8647 (16%), 7555 (14%) and 6711 (12%), respectively (Figure 3). Although ‘overdose’ had the highest number of total reports across all 21 drugs in the study, it is noteworthy to add that ‘toxicity to various agents’ emerged as the most reported reaction for six specific drugs (xylazine, pentobarbital, carfentanil, levamisole, furosemide, and amitriptyline). In these cases, this reaction had the highest number of reports among all reactions associated with those drugs individually.

### 3.5. Polysubstance Use

**Figure 4:**
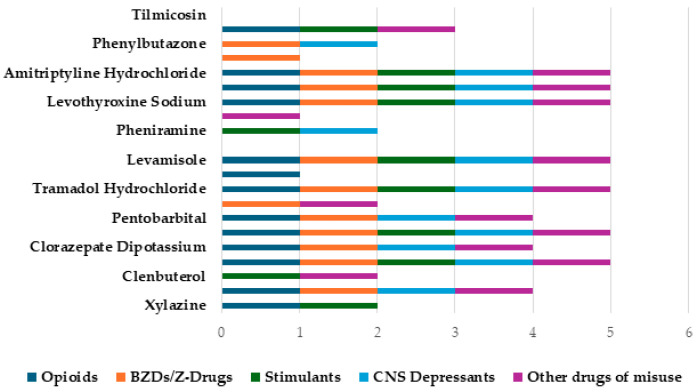
A stacked bar chart showing polysubstance use.

Out of all the drugs included in this study, 90% had reports of co-use with other drugs of misuse (19/21), and BZDs/Z-Drugs and opioids were implicated in AEs associated with 62% (13/21) of drugs analysed. Stimulants were implicated in AEs associated with 57% (12/21), CNS depressants in 57% (12/21), and 67% of the drugs under study were found to have been used concomitantly with drugs of misuse categorised as ‘other’. Figure 4 is a visual representation of the concurrent use of other drugs of misuse alongside the drugs of interest investigated in this study.

### 3.6. Reactions Associated with Animal-Only Drugs

Quantitative analysis was performed on the reactions associated with the eight drugs exclusively approved for animal use (xylazine, levamisole, pentobarbital, carfentanil, phenylbutazone, acepromazine, tilmicosin, and carprofen), revealing a total of 27 reactions through unmasking techniques. Carfentanil, acepromazine, tilmicosin, and carprofen did not have any data. Using a frequency analysis of AEs identified through unmasking techniques, this analysis quantified the number of distinct adverse reactions reported for each drug, focusing on reactions that were isolated from cases involving other drugs. This ensured that the reported effects were attributed solely to the drug of interest, excluding any influence from the concurrent use of other drugs. The unmasking of these drugs revealed that ‘intentional overdose’ and ‘overdose’ were the most reported reactions associated with these drugs when taken alone, with seven reports each (26%). Other reactions included ‘accidental overdose’ with four reports (15%), and ‘toxicity to various agents’ with three reports (11%). ‘Completed suicide’ received two reports (7%) and ‘intentional product misuse’, ‘withdrawal syndrome’, ‘drug withdrawal syndrome’, and ‘suicide attempt’ all received one report (4%) each. For the eight animal-only drugs, 63% (5 out of 8) had ‘died’ as the most common outcome, these drugs included xylazine (81 reports, 74%), levamisole (280 reports, 71%), carfentanil (92 reports, 84%), tilmicosin (1 report, 100%), and carprofen (12 reports, 86%). For the eight drugs licenced for animals only, further analyses was conducted and is summarised in Table 1.

## 4. Discussion

This article examined the FAERS AEs related to veterinary drug misuse, specifically, the 21 drugs identified from the SLR [23]. With the increasing misuse of prescription drugs being described as a modern epidemic [29], veterinary prescription drugs are also becoming more apparent in the illicit drug market. Their rising use can be associated with the lack of stringent regulations, easy access, and low cost, rendering them an appealing option for misuse. The analysis confirmed that veterinary medicines can be subject to diversion, misuse, and dependence, as well as be used for acts of suicide. The growing body of evidence and rising number of reports on the misuse and abuse of veterinary drugs highlight this area as a significant cause of concern, which lacks current, up-to-date research. A total of 38,756 cases from the FAERS were identified for the 21 drugs detected in the systematic literature search conducted before this study. To our knowledge, this is the first study utilising the FAERS database to gain data regarding the growing misuse of veterinary products.

The results of this study reveal a significant increase in the number of AE reports associated with veterinary products. When analysing the groups of drugs, it was found that the year 2023 accounted for over one-third (14,540, 33%) of the total reports across the last decade (43,555). For the 21 drugs analysed, 9 (ketamine, clenbuterol, butorphanol, tramadol, levamisole, stanozolol, levothyroxine, furosemide and amitriptyline) exhibited an increase of over 1000% in reports over the past five years, highlighting a significant rise in potential misuse and increased awareness and reporting. Additionally, six drugs demonstrated a percentage increase between 500 and 999% (dexmedetomidine, clorazepate, phenobarbital, pentobarbital, carfentanil and phenylbutazone) over the same period. Reports of pheniramine rose by 300%, xylazine by 136%, and acepromazine by 21%. The remaining three drugs (dinoprost, carprofen and tilmicosin) did not show any percentage increase over the last 5 years, suggesting a potentially low risk of misuse. However, given that these drugs are primarily intended for veterinary use, human reports are inherently low as they are not commonly prescribed drugs in human medicine.

### 4.1. Drugs Approved for Animals Only

Eight drugs within the study are exclusively licenced for animal use only (xylazine, pentobarbital, carfentanil, levamisole, acepromazine, tilmicosin, carprofen, and phenylbutazone). Notably, a recent report [11] highlights the presence of xylazine, pentobarbital, and levamisole in counterfeit fentanyl tablets. Furthermore, research shows that carfentanil, a fentanyl analogue, is increasingly prevalent in opioid overdose deaths [30]. These instances underscore the growing use of veterinary products as adulterants in illicit drugs. The FAERS database primarily focuses on human products that have been approved and licenced by the FDA. Although it does include data on unapproved products, data regarding veterinary products are sparse. The inclusion of data on animal-only drugs in the FAERS database indicates a noticeable rise in their usage, as individuals and healthcare reporters are increasingly documenting adverse effects linked to these medications. Two animal-only drugs (carprofen and tilmicosin) did not receive any reports related to the specific PTs in the last decade.

Examining the remaining six drugs approved exclusively for animal use, xylazine, levamisole, and pentobarbital have shown an upward trend in reports up until 2023. Xylazine’s upward trend correlates with its increasing presence in illicit drug markets in the US, with the UK now demonstrating a small rise in cases also. In 2022, 98% of xylazine-related deaths involved fentanyl [31], leading to the US recognising fentanyl-associated xylazine as an emerging threat [15]. Similarly, the UK’s Advisory Council on the Misuse of Drugs (ACMD) released a statement in 2024 advising xylazine to be controlled with increased vigilance and monitoring, after evidence of xylazine detection in 16 people with 11 fatalities [17]. After its first reports to the FDA in 2021 (49 reports), there have been an additional 67 reports relating to the PTs specific to this study. The majority of xylazine’s

Total reports (87%) are for the specific PTs, indicating a significant number of reports related to misuse. The main motivations behind xylazine misuse are not fully understood, and most users do not intentionally seek out xylazine [32]. However, one study found a group of people who intentionally sought after xylazine and its desirable effects, including a prolonged duration of the high when taken with opioids [32].

Over the last 10 years, there have been 550 reports of levamisole use, with a significant spike in 2023 with 195 reports, marking a dramatic increase from the previous year’s 55 reports. The high reports for levamisole correlate to its prevalence in cocaine samples, where the Drug Enforcement Agency (DEA) stated that 87% of cocaine bricks contained the veterinary anti-helminthic [33]. Cocaine is the second most illicitly used drug after cannabis [34], and its frequent adulteration with levamisole has become a public health concern. This contamination poses serious risks, including vasculitis, a dangerous condition that can lead to organ failure as a result of levamisole poisoning [35]. Its various pharmacological properties contribute to its use as an adulterant, as it enhances neurotransmitter release by acting on dopamine, serotonin, and norepinephrine transporters. Additionally, it extends the effects of cocaine and influences neurotransmitter reuptake after cocaine is metabolised [36].

Since 2013, there has been a fluctuation in the number of reports concerning the barbiturate, pentobarbital. However, since 2021, there has been a constant increase, with the number of reports rising from 36 (2021) to 90 (2023). Notably, 2023 saw the highest number of FDA reports regarding pentobarbital to date, where an increase in reports could be associated with its rising identification of counterfeit fentanyl samples in the US [11]. Although historically used in humans to treat insomnia and manage seizures, pentobarbital’s main uses are as a euthanasia agent in veterinary medicine. Reports suggest that media coverage describing pentobarbital as a peaceful method of suicide [37] has led to increased interest in acquiring the drug from regions where it is less regulated. These reports align with findings from this study, where pentobarbital had 48 reports of ‘completed suicide’, this being its third most reported reaction after ‘off-label use’ (75 reports) and ‘toxicity to various agents’ (69 reports).

In contrast, carfentanil, acepromazine, and phenylbutazone have exhibited relatively fewer reports in 2023 (three, one, and one report, respectively). Carfentanil’s initial spike in reports, from 0 in 2016 to 23 in 2017, aligns with findings indicating that carfentanil ranked as the second most frequently reported synthetic opioid in the US between 2016 and 2017, and it was the most frequently seized drug among synthetic opioids in 2017 for the years 2015–2018 [38]. Additionally, in 2017, the World Health Organisation (WHO) recommended that carfentanil be moved to the most stringent level of international control due to the high potential for harm and dependence [39]. Although recent reports to the FDA may be low for carfentanil, it was documented that there is growing evidence that “carfentanil may be making a comeback”, as there was a 3400% increase in detection from 2022 to 2023 [40]. Given the extreme potency of this medication, it is crucial to allocate attention and effort to prevent an increase in reported cases.

Furthermore, The Centre for Forensic Science Research and Education (CFSRE) predicted acepromazine (phenothiazine) and phenylbutazone (a non-steroidal anti-inflammatory drug (NSAID)) as the ‘next xylazine’, emphasising the need for monitoring and testing due to their use as toxic adulterants, albeit the relatively low number of cases [41]. The CFSRE also reported that there were 116 seized drug samples from Pennsylvania between 2016 and 2021 containing phenylbutazone, raising concerns about its potential nationwide spread, mirroring the pattern observed with xylazine [42]. These samples predominantly included heroin, fentanyl, and xylazine. There is limited data available regarding the misuse of phenylbutazone, but there were 32 cases related to this study’s PTs, with 32 reports of overdose (36%) and 4 reports of completed suicide (12%). It is known to be a very potent NSAID with serious adverse effects on human health, including gastrointestinal bleeding, liver and kidney damage, and blood disorders [42]. Acepromazine has no approved use for humans and like phenylbutazone, data regarding its misuse is sparse. There were 31 cases retrieved for acepromazine, with 16 reports of overdose (41%), 7 cases of drug abuse (18%), and 3 cases of completed suicide (8%). It has been reported that CNS and respiratory depression are possible if ingested by humans [41]. Despite the relatively low cases of phenylbutazone and acepromazine, recent detection and a lack of scheduling means monitoring is warranted.

### 4.2. Demographics and Reporting of Drug AEs

Our analysis revealed notable disparities in the distribution of reported AEs among countries. The US exhibited the highest number of reported AEs (13,532), followed by France (3459), Canada (2869) and the UK (1400). These findings may reflect differences in veterinary medication usage patterns, regulatory practises, and healthcare reporting requirements across counties. The high number of reports from the US is consistent with expectations, given that the FAERS is a US-based database. Additionally, the significant prevalence of substance use disorders (SUD) in the US, with 46.8 million Americans (over age 12) battling SUD in 2022, underscores the widespread nature of the issue. Reports associated with the eight animal drugs were documented by 18 different countries, underscoring the emerging widespread, global problem of misuse of animal medications worldwide.

For every drug included in the whole FAERS database, females contributed to over 5 million more AE reports than males. This aligns with findings that female AE reports outnumber male AE reports across the world, in all age groups, although male reports more often contain more serious and fatal reports [43]. UK statistics in 2022 also demonstrated this pattern, where there were 1143.3 drug-related deaths registered per million among males (3240 deaths), compared with 55.8 deaths per million among females (1667 deaths) [2]. These statistics correspond to reports stating males have higher rates of use/dependence on illicit drugs than females [44] and males die from overdose at an approximately 2–3 times greater rate than females for opioids and stimulants [45]. In this study, although 57% (12/21) of drugs had more cases from males, the overall total of reports had slightly more from females (50%). For all drugs in this study, there was a higher number of deaths associated with females with 4529 deaths reported (53%), with 4005 death reports attributed to males (47%).

In the UK for the last 25 years, the age group with the highest rate of drug misuse deaths were those aged 40–49 [2]. As expected, the age group with the highest number of reports associated with the selected PTs were 18–64 years, contributing 14,766 (68%) of reports. Worryingly, 1772 reports were from children under 18 (8%), with 893 (4%) reports from those aged 0–11 years. It is unknown how these children gained these drugs and whether exposure was accidental or intentional. Various physiological differences between children and adults lead to significant variations in pharmacokinetics and pharmacodynamics, including gastric pH, first-pass metabolism, renal clearance, protein binding, protein concentration, and enzyme activities [46]. In 2023, the Office of National Statistics (ONS) reported 2734 drug poisonings from the years 1993–2022 for those aged under 20, with 1562 (57%) of these reports being classified as drug misuse [2].

‘Overdose’ emerged as the most common reaction reported, with 8647 reports (16%), suggesting a clinical public health concern associated with increasing misuse of these products. The high overdose rates could be linked to the potency of these medications, as drugs approved for animals will be tailored to their differing pharmacology. For example, carfentanil and phenylbutazone, two veterinary-only products, received 15 and 12 overdose reports, respectively. Carfentanil lacks approved medical applications in human healthcare settings. Its reported cases of overdose may stem from the considerable challenge of accurately dosing the substance [47]. Additionally, limited data on appropriate dosage schedules and abuse potential contribute to the ambiguity surrounding its usage [21]. Overdose emerged as the predominant report for phenylbutazone, possibly due to its toxic properties, leading to it being discontinued from human use after reports of death [42].

A higher percentage of reports coming from healthcare professionals (53%) indicates these drugs could have a higher risk, requiring medical intervention. Consumer reports may be slightly lower due to multiple reasons. Individuals who frequently misuse drugs or have SUD may have acquired them illegally, leading to a reluctance to report AEs out of fear of legal repercussions, this fear potentially acts as a deterrence to engage with formal reporting systems. Additionally, they may be hesitant to disclose information about their history of drug use, further reducing their likelihood of reporting AEs.

The prevalence of hospitalisation as the highest outcome underscores the severity of the harms and risks associated with veterinary medication. The high rate of hospitalisation (44%) suggests these drugs have a high toxicity profile, which could be due to the differences in dosing between human and animal medicine, as well as differences in metabolism and tolerance. Certain veterinary medications, which are also approved for humans, may vary in dosage. For instance, veterinary ketamine formulation can be ten times stronger than medicinal ketamine for humans [48]. For medicines approved for animals only, dosages can be increasingly more potent for human drugs within the same class, such as the differences in potency between carfentanil and morphine, with carfentanil being 10,000 more potent than morphine [49].

Worryingly, death was the second most reported event for the drugs included in this study, with 9566 reports (34%). Unfortunately, it remains uncertain whether these fatalities resulted from intentional or accidental actions, and the specific dosages involved are also unknown. Hospitalisation and death rates may also be associated with the increasing adulteration using veterinary medication. As adulteration increases, users are more exposed and are at increased risk of harm as they are ingesting multiple drugs that can have additive or synergistic effects. When xylazine is combined with fentanyl or other synthetic opioids, xylazine can increase the potential of fatal overdoses, due to increased respiratory depression [16]. Adulteration of drugs using veterinary medication is increasing, where more potent drug mixtures are being identified and illicit drug production in Europe continues to grow [14]. Individuals struggling with drug addiction may resort to seeking more potent substances to fulfil their cravings, and turning to veterinary medications is the way drug manufacturers are targeting these problematic users.

### 4.3. Polydrug Use

The simultaneous consumption of multiple drugs is a common behaviour among many PWUDs and was a trend observed within this study also. SUD with polysubstances has been identified as a significant factor in the public health crisis of overdose toxicity [50], where it may be utilised to enhance a drug’s effects, where additive or synergistic effects are often desired. Xylazine is infrequently found on its own and is rarely the primary purchase purpose of buyers [32]; nonetheless, data have demonstrated that it is becoming more common in drug samples as a co-detected drug. Recent data from the ACMD presented that 100% of xylazine detection in the UK also included other drugs of misuse, with most samples including more than six other drugs, including heroin, cocaine, bromazolam, fentanyl, ketamine, metonitazene, and protonitazene [17]. The Welsh Emerging Drugs and Identification of Novel Substances (WEDINOS) have received samples of xylazine from 2020 and since then they have received 48 samples, where 100% were not the buyer’s purchase intent. Of these, 31 (65%) samples contained two or more other substances including synthetic opioids (metnitazene) and designer BZDs (bromazolam). Alarmingly, there were two cases of a tetrahydrocannabinol (THC) vape containing xylazine [51]. The polysubstance use of xylazine with other drugs of misuse exacerbates the risk of overdose and fatalities, where increased CNS depression and respiratory depression are often observed. Moreover, the clandestine nature of xylazine’s presence in drug samples, often without the knowledge of the user, can further complicate the diagnosis and understanding of a patient’s conditions. While naloxone, an opioid antagonist, has been deemed ineffective in reversing the effects of xylazine [16], it is recommended for administration due to the frequent co-occurrence of xylazine with opioids [52]. However, recent research utilising rat models has reported xylazine to be a full kappa-opioid receptor agonist and was shown to be responsive to the antidote naloxone [53]. Although xylazine’s role in opioid-induced deaths, in a polysubstance use context, is largely unknown, one report shows a connection between xylazine and opioid co-use and its effects on brain oxygenation and brain temperature [54]. This suggests that xylazine may exacerbate the life-threatening effects of opioids by worsening brain hypoxia. Polysubstance use data in this study revealed that 90% (19/21) of drugs analysed had reports of concurrent use with common drugs of misuse, where BZDs/Z-drugs and opioids were the most popular. Interestingly, it was discovered that the veterinary drugs we examined were also co-used with other veterinary drugs included in this study. For example, there were reports of ketamine being used alongside dexmedetomidine, clenbuterol, phenobarbital, and pentobarbital. Additionally, pentobarbital was co-used with dexmedetomidine, and there was one report of levamisole with carfentanil. Other common drugs of misuse were also commonly reported alongside the drugs of interest, including cocaine, heroin, alprazolam, pregabalin, fentanyl, flunitrazepam, and etizolam. This high level of polysubstance misuse can directly relate to significant risks to PWUDs, where there are increased risks of overdose and severe medical complications. The increasing rate of using veterinary compounds as adulterants is of significant concern to public health as testing for these products is extremely limited, leading to treatment that is not precise and accurate for the patient’s specific needs. This can lead to challenges in managing overdose events and underscores the importance of expanding testing capabilities and increasing awareness among healthcare providers about the emerging presence of veterinary products as adulterants. Notably, within this study, overdose emerged as the most prevalent reported reaction, further highlighting the urgency of these measures.

The current gaps in veterinary drug misuse highlight the need for more comprehensive research, where research is needed to better understand the health impacts, prevalence, and patterns of this type of drug misuse. Expanding control measures to include a broader range of veterinary drugs could mitigate misuse and improve monitoring, where policymakers should consider implementing policies that address the growing trend in veterinary drug misuse. Increased awareness, including educational campaigns to target the general public and high-risk groups of PWUDs, could be utilised to disseminate important information to reduce misuse and associated harm. Future work recommendations should focus on further exploration of under-reported, uncontrolled veterinary drugs, aiming to explore their misuse potential and any associated health effects. This can inform healthcare professionals of potential emerging drugs before they rise in prevalence. Further laboratory research is needed to enhance the understanding of the pharmacological properties of these drugs in humans, as existing data predominantly pertain to their use in animals.

## 5. Conclusions

This descriptive pharmacovigilance study aimed at analysing the adverse event reports associated with drugs commonly used in veterinary medicine. The FDA database is a human-drug reporting system; therefore, the reporting of AEs of animal-only medications is alarming and demonstrates an increasing misuse rate. Similar to previous studies utilising the FAERS database to analyse substance misuse [55], the FAERS tool remains an important pharmacovigilance tool to identify early safety signals and to gain valuable data associated with emerging drugs of misuse. This method of investigating veterinary drug use is important as instances of veterinary medication misuse remain largely underreported. This analysis reveals a rising trend in veterinary product misuse and its associated reporting, as indicated by the year 2023 accounting for approximately one-third of all reports for these products during the previous decade. Socio-demographic findings showed that overall, for all 21 drugs, females had a slightly higher number of reports than males, the age group 18–64 demonstrated the highest number of total reports, and healthcare professionals contributed to 53% of all reports. Hospitalisation was the most common outcome reported, and non-serious outcomes only accounted for 5% of cases, although death was the most common outcome when observing animal-only products. Overdose was the most reported reaction overall, yet unmasking techniques of the drugs exclusively approved for animals only revealed that intentional overdose was the most reported reaction. Veterinary medicines are increasingly infiltrating the illicit drug market due to their potent pharmacological effects, highlighting the need for increased vigilance to address this emerging public health concern. It is crucial to raise awareness of this issue to ensure all healthcare professionals are equipped with the necessary knowledge to address the growing use of veterinary products in the illicit drug market. It is also pivotal that PWUDs are aware of the risks associated with this type of drug consumption, particularly with the growing prevalence of adulteration involving veterinary products.

## 6. Limitations

While the findings of this study offer valuable insights into the misuse of veterinary medicines, there are limitations present that must be acknowledged. The FAERS database is a spontaneous reporting system that, despite its global reach, inherently suffers from underreporting, incomplete case information, and potential bias towards the reporting of only severe or unexpected adverse events [56]. Pharmacovigilance disproportionality analyses are influenced by various biases, such as the Weber effect, which leads to a spike in reporting adverse drug reactions soon after a drug is launched. This happens due to limited initial safety data and increased exposure. These analyses also struggle to identify long-term effects as they move further from the drug’s launch. Media coverage of an adverse reaction can further drive-up reporting, known as the notoriety effect. Additionally, patient-related biases can skew comparisons in the data. These biases, along with issues of underreporting, highlight the need for careful interpretation and the use of alternative approaches in pharmacovigilance research [57]. Due to FAERS being a US-based database, the findings of this study cannot be generalised to regions outside of the US. It is also important to note that changes in reporting practises, such as increased awareness or regulatory changes, might lead to spikes in reports that do not necessarily correlate with actual increases in misuse or AEs. Other limitations of the FAERS include the broad age range classification (18–64), which complicates the reporting and analysis of age-specific demographics, as well as the lack of detailed information on factors such as dosages and routes of administration. Due to the FAERS being a human-based reporting system, data for specific drugs (e.g., medetomidine) were not available to analyse. The focus of the study was veterinary medicines, meaning formulations of drugs (e.g., intranasal ketamine) were also not included in the study. Specific statistical analysis, such as the ROR, could also not be conducted for this analysis due to the drugs’ diverse classification across different drug classes. As the drugs belonged to varying drug groups, comparing their adverse event reporting frequencies may not yield meaningful results.

## Figures and Tables

**Table 1:** Reports for drugs licenced for animal use only.

Drugs	ADRs Associated to PTs	Most Common Reactions	Gender	Age	Reporter	Outcome	Concomitant Drugs
Xylazine	95 (87%)	Toxicity to Various Agents = 68 (58%) Drug Abuse = 26 (22%) Overdose = 9 (8%)	F = 20 (30%) M = 67 (77%)	18–64 = 74 (91%) 3–11 = 5 (6%)	Healthcare professional = 90 (95%) Consumer = 5 (5%)	Died = 81 (74%) Hospitalisation = 16 (15%) Life Threatening = 9 (8%)	Opioids = morphine, codeine, tramadol, methadone. Stimulants = amphetamine, cocaine
Levamisole	372 (73%)	Toxicity to Various Agents = 216 (37%), Drug Abuse = 134 (23%) Completed Suicide = 48 (8%)	F = 94 (28%) M = 236 (72%)	18–64 = 305 (95%) 12–17 = 10 (3%)	Healthcare Professional = 334 (95%) Consumer = 17 (5%)	Died = 280 (71%) Hospitalised = 87 (22%) Life Threatening = 25 (6%)	Opioids = carfentanil, morphine. Stimulants = caffeine, cocaine, nicotine. BZDs/Z-drugs = diazepam, temazepam. CNS depressants = alcohol. Others = gabapentin, pregabalin
Pentobarbital	220 (45%)	Off Label Use = 75 (27%), Toxicity to Various Agents = 69 (25%) Completed Suicide = 48 (17%)	F = 114 (56%) M = 90 (44%)	18–64 = 126 (65%) 3–11 = 30 (15%) 12–17 = 16 (8%) 2 month-2 = 10 (5%)	Healthcare professional = 202 (95%) Consumer = 11 (5%)	Died = 109 (37%) Hospitalised = 113 (39%) Life Threatening = 52 (18%)	Opioids = fentanyl, tramadol, loperamide. BZDs/Z-drugs = diazepam, oxazepam, nordazepam, valium, clonazepam, lorazepam, clobazam, flunitrazepam, midazolam, dalmane, zolpidem. CNS Depressants = phenobarbital, dexmedetomidine. Others = ketamine, gabapentin, dextromethorphan
Carfentanil	104 (88%)	Toxicity to Various Agents = 79 (54%) Drug Abuse = 27 (19%), Overdose = 15 (10%)	F = 48 (48%) M = 52 (52%)	18–64 = 97 (99%) 12–17 = 1 (1%)	Healthcare professional = 90 (95%) Consumer = 5 (5%)	Died = 92 (84%) Hospitalised = 15 (14%) Life Threatening = 3 (3%)	Opioids = codeine
Phenylbutazone	32 (3%)	Overdose = 12 (36%) Intentional overdose = 5 (15%) Accidental overdose = 4 (12%) Completed suicide = 4 (12%)	F = 12 (41%) M = 17 (59%)	18–64 = 18 (75%) 12–17 = 2 (8%) 65–84 = 2 (8%)	Healthcare Professional = 19 (59%) Consumer = 13 (41%)	Died = 10 (40%) Hospitalised = 12 (48%) Life Threatening = 3 (12%)	BZDs/Z-drugs = diazepam. CNS depressants = alcohol
Acepromazine	31 (74%)	Overdose = 16 (41%) Drug Abuse = 7 (18%) Intentional Overdose = 3 (8%) Completed Suicide = 3 (8) Suicide Attempt = 3 (8%)	F = 19 (66%) M = 10 (34%)	18–64 = 29 (100%)	Healthcare Professional = 30 (100%)	Died = 9 (20.45%) Hospitalised = 21 (48%) Life Threatening = 14 (32%)	No results
Tilmicosin	1 (100%)	Completed Suicide = 1 (100%)	F = 1 (100%)	18–64 = 1 (100%)	N/A	Died = 1 (100%)	BZDs/Z-drugs = Zopiclone
Carprofen	12 (50%)	Completed Suicide = 8 (53%) Toxicity to Various Agents = 7 (47%)	F = 12 (100%)	18–64 = 12 (100%)	Healthcare Professional = 10 (100%)	Died = 12 (85.71%) Hospitalised =2 (14%)	Opioids = Hydromorphone. Stimulants =Amphetamine sulphate. Others = Promethazine

## Data Availability

Data supporting reported results can be made available upon request.

## Supplementary Materials

The following supporting information can be downloaded at: https://www.mdpi.com/article/10.3390/toxics12110777/s1, Table S1: A list of commonly misused drugs, including brand names, used to analyse concomitant drug use.
