# Supplementary material for: Exploring Human Misuse and Abuse of Veterinary Drugs: A Descriptive Pharmacovigilance Analysis Utilising the Food and Drug Administration’s Adverse Events Reporting System (FAERS)
Source: Toxics. 2024 Oct 25;12(11):777. doi: 10.3390/toxics12110777 (PMC11597946; doi:10.3390/toxics12110777)
Supplement: Supplementary file 1 [file toxics-12-00777-s001.zip › toxics-3214467-supplementary.pdf]

Supplementary Table S1 - A list of commonly misused drugs, including brand names, used to analyse concomitant drug use

Opioids	Benzodiazepines/Z-drugs	CNS Depressants	Stimulants	Other
Fentanyl	Adinazolam	Xylazine	Caffeine	Ketamine
Morphine	Deracyn	Dexmedatomidine	Nicotine	Ketamine HCl
Hydrocodone	Alprazolam	Medetomidine	Cocaine	Ketamine Hydrocloride
Methadone	Xanax	Pentobarbital	Amphetamine	Cannabis
Oxycodone	Helex	Phenobarbital	Concerta	Marijuana
Carfentanil	Xanor	Alcohol	Ritalin	LSD
Buprenorphine	Trankimazin	Ethanol	Methylphenidate	Mescaline
Hydromorphone	Onax	Nembutal	Adderall	PCP
Codeine	Alprox	Gamma-hydroxybutyrate	Methamphetamine	Psilocybin
Heroin	Misar	GHB	Dextroamphetamine	Salvia
Oxymorphone	Restyl	Sodium oxybate	Khat	Ayahuasca
Anexsia	Solanax	Xyrem	Kratom	Amyl nitrate
Co-Gesic	Tafil	Acepromazine	MDMA	Ketalar
Embeda	Neurol		Desoxyn	Spravato
Exalgo	Frontin		Clenbuterol	Ketaset
Hycet	Kalma			Dextromethorphan
Hycodan	Ksalol			Nandrolone
Hydromet	Farmapram			Oxandrin
Ibudone	Bentazepam			Oxandrolone
Kadian	Thiadipona			Anadrol
Liquicet	Bretazenil			Oxymetholone
Lorcet	Bromazepam			Anadrol-50
Lortab	Lexotanil			Testosterone cypionate
Maxidone	Lexotan			Depo-testosterone
MS Contin	Lexilium			Gabapentin
Norco	Lectopam			Levothyroxine
Opana	Lexaurin			Levothyroxine Sodium
OxyContin	Lexatin			Furosemide
Palladone	Bromam			Amitriptyline
Percocet	Bromazolam			Phenylbutazone
Percodan	Brotizolam			Carprofen
Reprexain	Lendormin			Pregabalin
Rezira	Dormex			Promethazine
Roxicet	Sintonal			Pheniramine
Targiniq	Noctilan			
TussiCaps	Camazepam			

Tussionex	Albego
Tuzlstra	Limpidon
Vicodin	Librium
Vicoprofen	Risolid
Vituz	Elenium
Xartemis	Cinazepam
Xodol	Levana
Zolvit	Cinolazepam
Zutripo	Gerodorm
Zydone	Clobazam
Actiq	Onfil
Duragesic	Frisium
Sublimaze	Urbanol
Zohyrdo	Clonazepam
Dilaudid	Rivatril
Meperidine	Rivotril
Demerol	Klonopin
Dolophine	Iktorivil
Methadose	Paxam
Duramorph	Clonazolam
Zohydro	Clorazepate
Tramadol	Tranxene
Tramadol HCl	Tranxilium
Butorphanol	Clotiazepam
Loperamide Hydrochloride	Veratran
	Clozan
	Rize
	Cloxazolam
	Cloxam
	Sepazon
	Olcadil
	Delorazepam
	Dadumir
	Chloretizolam
	Diazepam
	Antenex
	Apaurin
	Apzepam
	Apozepam
	Diazepan
	Hexalid

	Normabel
	Pax
	Stesolid
	Stedon
	Tranquirit
	Valium
	Vival
	Valaxona
	Diclazepam
	Estazolam
	Ethyl carfluzepate
	Etizolam
	Etilaam
	Etizest
	Pasaden
	Depas
	Ethyl lofazepate
	Victan
	Meilax
	Ronlax
	Flualprazolam
	Flubromazepam
	Templex
	Flubromazolam
	Remnon
	Flubrotizolam
	Fluclotizolam
	Flunitrazepam
	Rohypnol
	Hipnosedon
	Vulbegal
	Fluscand
	Flunipam
	Ronal
	Rohydorm
	Hypnodorm
	Flunirtazolam
	Fluazepam
	Dalmadorm
	Dalmane
	Fluzepam
	Flutazolam

	Coreminal
	Flutemazepam
	Flutoprazepam
	Restas
	Halazepam
	Alapryl
	Paxipam
	Ketazolam
	Anxon
	Sedotime
	Loprazolam
	Dormonoct
	Havlane
	Lorazepam
	Atvian
	Orfidal
	Lorenin
	Lorsailan
	Temesta
	Tavor
	Lorabenz
	Lormetazepam
	Loramet
	Noctamid
	Pronoctan
	Meclonzepam
	Medazepam
	Nobrium
	Ansilan
	Mazepam
	Rudotel
	Raporan
	Metizolam
	Mexazolam
	Midazolam
	Dormicum
	Flormidal
	Versed
	Hypnovel
	Dormonid
	Nifoxipam
	Nimetazepam

	Erimin
	Lavol
	Nitemazepam
	Nitrazepam
	Mogadon
	Alodorm
	Pacisyn
	Dulmolid
	Nitrazadon
	Nitrazolam
	Nordazepam
	Norfluazepam
	Oxazepam
	Seresta
	Serax
	Serenid
	Serepax
	Sobril
	Oxabenz
	Oxapax
	Oxascand
	Ox-Pam
	Opamox
	Alepam
	Medopam
	Murelax
	Noripam
	Purata
	Phenezepam
	Phenzitat
	Pinazepam
	Domar
	Duna
	Prazepam
	Demetrin
	Lysanxia
	Prazene
	Centrax
	Premazepam
	Pyrazolam
	Quazepam
	Doral

	Quiedorm
	Rilmazefone
	Temezepam
	Restoril
	Normison
	Euuhypnos
	Temaze
	Tenox
	Tetrazepam
	Myolastan
	Clinoxam
	Epsipam
	Musaril
	Triazolam
	Halcion
	Rilamir
	Notison
	Somese
	Flumazenil
	Anexate
	Lanexat
	Romazicon
	Mazixcon
	Eszopiclone
	Lunesta
	Zaleplon
	Sonata
	Starnoc
	Zolpidem
	Ambien
	Nytamel
	Snaval
	Stilnoct
	Stilnox
	Sublinox
	Xolnox
	Zoldem
	Zolnod
	Zopiclone
	Imovane
	Rhovane
	Ximovan

	Zileze
	Zimoclone
	Zimovane
	Zopitan
	Zoeclone
	Zopiklone
	Lunesta
	Atvian
	Clorazepate Dipotassium
	Chlordiazepoxide
